# Factors associated with stunting among children of age 24 to 59 months in Meskan district, Gurage Zone, South Ethiopia: a case-control study

**DOI:** 10.1186/1471-2458-14-800

**Published:** 2014-08-07

**Authors:** Teshale Fikadu, Sahilu Assegid, Lamessa Dube

**Affiliations:** Arba Minch Health Science College, Arba Minch, Ethiopia; College of Public Health and Medical Sciences, Department of Epidemiology, Jimma University, Jimma, 378 Ethiopia

**Keywords:** Stunting, Children, Factors, Ethiopia

## Abstract

**Background:**

Stunting is one of the major causes of morbidity among under-five children Knowledge about risk factors of stunting is an important precondition for developing and strengthening nutritional intervention strategies. The purpose of this study was to assess factors associated with stunting among children of age 24 to 59 months in Meskan District of Gurage Zone, South Ethiopia.

**Methods:**

Community based case-control study was conducted among children of age 24 to 59 months. A multistage sampling technique was used to select the study participants. Cases were stunted children while controls were not stunted children. A total of 121 cases and 121 controls were studied.. Data were analyzed using SPSS 16.0 statistical software.

**Results:**

Children living in households with eight to ten [Adjusted Odds Ratio (AOR) = 4.44, 95% CI: 1.65, 11.95] and five to seven [AOR = 2.97, 95% CI: 1.41, 6.29] family members were more likely to be stunted than those living in households with two to four family members. Similarly, children living in households with three under-five children [AOR = 3.77, 95% CI: 1.33, 10.74] were more likely to develop stunting than those living in households with one under-five child. Children whose mothers worked as merchants [AOR = 4.03, 95% CI: 1.60, 10.17] were more likely to be stunted than children whose mothers worked as house wives. Children who breast fed for <2 years [AOR = 5.61, 95% CI: 1.49, 11.08] were more likely to be stunted than those who breast fed ≥2 years. Children who were exclusively breast fed for <6 months [AOR = 3.27, 95% CI: 1.21, 8.82]were more likely to develop stunting than children who were exclusively breast fed for the first 6 months. Children who bottle fed [AOR =3.30, 95% CI: 1.33, 8.17)] were more likely to be stunted than children who fed their complementary food using spoon/cup.

**Conclusions:**

Family size, number of under-five children in the household, maternal occupation, duration of exclusive breastfeeding, duration breast feeding, and method of feeding complementary food were independently associated with stunting. Thus, public health intervention working on improving child nutrition should consider these determinants.

## Background

Adequate provision of nutrients is crucial to ensure good physical & mental development as well as for long-term health. Undernutrition accounts 35% of all deaths among under-five children. More than 2 million under-five children die each year due to undernutrition [1]. Undernutrition refers to a state resulting from a relative or absolute deficiency of one or more essential nutrients. The three main indicators used to define undernutrition, i.e., stunting, underweight and wasting; that represent different histories of nutritional insult to the child and measured by height for age, weight for height and weight for age indexes respectively. Stunting is a measure of chronic malnutrition. Undernutrition in low and middle-income countries was high. Both stunting & underweight are highest in Eastern Africa. About 42% of children in sub-Saharan Africa and 182 million (32.5%) children in developing countries were undernourished and the stunting trend was stagnant in Africa from1990 to 2010 [2–6].

Undernutrition can best be described in Ethiopia as a long term year round phenomenon due to chronic inadequacies of food combined with high levels of illness in under-five children which 44.0%, 29.0% & 10.0% were stunted; underweight and wasted respectively [7]. Similarly 53.6%, 32.7% & 7.0% of children aged 24 to 59 months were stunted, underweight and wasted respectively [7].

Studies show that stunting is associated with deprived attention, memory impairment, reduced learning, and memory in children, low school enrollment, and decreased higher cognitive functioning with a slowing in the rate of cognitive development. These finally result in low adult wages and lost productivity [8–10].

The causes of malnutrition are numerous and multifaceted. These causes are intertwined with each other and are hierarchically related. The most immediate determinants are poor diet and disease which are themselves caused by a set of underlying factors; household food security, maternal/child caring practices and access to health services and healthy environment. These underlying factors themselves are influenced by the basic factors (socio-economic and political conditions) [2, 11].

Undernutrition are multiple and inextricably linked to poverty (low socioeconomic status). Many studies revealed that lowest prevalence of stunting was found among children from families with higher per capital income (socioeconomic status) [12–14]. When a child lives in an area of low income level the chance of disease increase and these diseases can increase the risk of stunting [15]. More children suffering from stunting were observed among mothers of low educational levels than those with high educational levels [13, 16, 17]. Under- five children living in a household with large family were at higher risk of being stunted. Similarly, under- five children living in households with more than one under-five were more likely to be stunted [12, 13, 17]. Studies conducted in Ethiopia and abroad reported that the quantity, frequency, and type of supplementary feeding, birth weight, sex, birth order and disease conditions like diarrhea were strongly associated with stunting among under-five children [13, 15, 18–30]. Housing environment such as lack of safe water supply, access to a toilet facility and inadequate sanitation were among the factors that increased the risk of stunting [20, 25].

Comprehensive knowledge about the risk factors of stunting in local context is vital to reduce stunting rate, to develop prevention strategies and strengthen nutrition intervention programs. Despite higher prevalence of stunting among children age 24 to 59 months, there are limited studies that reports about risk factors of stunting among children age 24 to 59 months separately. Besides neither case-control nor cohort study was done to identify risk factors of stunting in the district. Thus the purpose of this study was to assess factors associated with stunting among children of age 24 to 59 months in Meskan district for the development and improvement of implementation and intervention strategies to reduce child mortality and morbidity.

## Methods

### Study setting, design and sampling

A community based case-control study was conducted from February to March 2013 in Meskan district, Gurage Zone, South Ethiopia. The district is located at 135 kilometers south of Addis Ababa *(the capital city of Ethiopia).* It had an estimated total population of 228,852 and under-five children of 35,724 from this about 61% (21970) were between the ages of 24 to 59 months in the year 2012/13 which is projected from 2007 Ethiopia Central Statistical Agency. The district has a total of 50 functioning health institutions (2 hospital 8 health center and 40 health posts).

Cases were stunted children aged 24 to 59 months: height-for age z- score below -2SD from the median height of the WHO reference population. While Controls were children aged 24 to 59 months without stunting.

Sample size was calculated using Epi info version 7 by assuming the proportion of mother with educational status of primary or less among controls and cases were 70.4% and 91.3% respectively [30], 95% CI, 80% power, case to control ratio of 1:1, design effect of 2 and accounted for 10% possible non-response. The total sample size was 242 (121 cases and 121 controls). To calculate sample size, maternal education was chosen as an independent variable since it gave maximum sample size and study conducted in Uganda was used because we did not find the proportions among cases and controls of there was neither a study done of children ages of 24-59 months in Ethiopia. Design effect was used. The reason is two steps were required to reach or identify the study participants.

A multistage sampling technique was used. Two urban and thirteen rural kebeles *(the smallest administration unit in Ethiopia)* were selected out of 6 urban & 40 rural kebeles by simple random sampling technique (lottery method) after stratifying the district in to urban and rural kebeles. All children of age 24 to 59 months living in selected kebeles were measured for their z-score of height for age and categorized as stunted and not stunted to generate sampling frames for cases and controls by a census conducted prior to the actual data collection. During the census 15,316 households were included from the 15 selected kebeles. A total of 7,367 (3,445 stunted and 3,922 not stunted) children between age of 24 to 59 months were measured for height for age. The total sample size of cases (stunted children) was allocated proportionally to the selected kebeles based on number of stunted children identified during census. Then simple random sampling method (generated by computer) was used to recruit cases from each selected kebele. A control was selected from the next house (neighbor) using a code of house number in ascending order. If two or more eligible controls were found in the same household then one of them was selected randomly.

### Measurements

Thirty health extension workers were trained to carry out a census to identify source population of cases and controls group, via interviewing the mother and obtaining the anthropometric measurements of each child. During the census health extension workers measured the height and identified the age of each child aged between 24 and 59 months. Height was measured in a standing-up position to the nearest 0.1 cm using a standard vertical board with a detachable sliding headpiece. In rural areas it is very difficult to get the age correctly. This may leads to misclassification of cases and controls. Thus ages of children were estimated using Expanded Program of Immunization registration book or immunization card when possible and asking the mother. The indices were calculated using WHO *Anthroplus* version 3.2.2 statistical software.

Data were collected using structured questionnaire via face to face interview with participant’s mothers or care takers. In this study the following independent variables or factors were assessed: religion, ethnicity, wealth index, family size, parental age, marital status, parental occupation, maternal knowledge, number of under-five children, source of drinking water, availability of toilet, waste disposal, antenatal care, place of delivery, immunization status, time of initiation of breast feeding, duration of exclusive breast feeding, duration of breast feeding, time of initiation of complementary feeding, type of complementary food, method of complementary feeding and number of meal per day, diarrhea, malaria and acute respiratory tract infections. In addition to these age, sex, type of birth (singleton or multiple), birth order of the child were also assessed. The questionnaire was initially prepared in English and translated into local language, then retranslated to check consistency. The instrument was pre-tested in 5% of sample size in non-selected kebele. The data were collected by five data collectors who have first degree in public health after two days training. Filled questionnaires were checked daily for its completeness by supervisors.

### Data analysis

Data were checked for completeness, edited, coded and entered into Epi data version 3.1 and exported to SPSS 16.0 statistical software for analysis. Frequencies and cross tabulations were used to check consistency. Composite scales were constructed to represent a single construct.

In this study current maternal knowledge as a proxy of past maternal knowledge was assessed by eleven knowledge questions on breast feeding (time of initiation, exclusive breast feeding and duration breast feeding) and complementary feeding practices (time of starting, method of feeding and frequency). If a mother responds correctly < 60%, 60%-75% and > 75% of the total knowledge questions, she is considered as having poor, fair and god knowledge respectively. Wealth index was computed as a composite indicator of living standard based on variables related to ownership of selected household assets, agricultural land, quantity of livestock and materials used for housing construction. The computation was made using principal component analysis and a single continuous variable was generated by summing up the principal components into one. Quartiles of wealth index were generated using the composite score.

After cleaning data for inconsistencies and missing values, descriptive statistics were done. Then after, bivariate analysis was done for all explanatory variables to identify those associated with children stunting. Variables with p-value less than 0.25 in the bivariate analysis were included in a backward stepwise logistic regression procedure. Odds ratios (95% confidence intervals) were calculated to determine the association between stunting and independent variables. Model fitness was assessed using Hosmer and Lemeshow test (p = 0.697). Collinearity and interaction between independent variables were checked and not found. Data were presented using tables.

### Ethical consideration

The ethical clearance was obtained from Jimma University, Ethical review board. Written consent was obtained from caretakers of under-five children. Child with no treated diarrhea, respiratory tack infection and fever/malaria were referred to nearby health centre.

## Results

A total of 242 children (121cases and 121 controls) were participated in this study. All of the study participants were singleton birth. Sixty five (53.7%) of cases as well as controls were male. The median birth orders of cases & controls were 4 and 3 respectively. Hundred thirteen (93.4%) of children’s mothers were Gurage by their ethnicity. Majority of participants’ mothers, 97(80.2%) of cases and 90(74.3%) of controls, were Muslim by religion. Mothers of 68(56.2%) and 59(48.8%) of cases and controls were illiterate respectively. Nearly two fifth of the controls’ mothers were house wife (Table 1).

**Table 1:** **Socio-demographic characteristics of the study participants and their mothers in Meskan district, Gurage Zone, South Ethiopia, March 2013**

Variable	Case control		p-value
	Nº (%)	Nº (%)	
Sex			0.89
Male	65(53.7)	65(53.7)	
Female	56(46.3)	56(46.3)	
Ethnicity			0.80
Gurage	113(93.4)	113(93.4)	
Other than Gurage	8(6.6)	8(6.6)	
Maternal Religion			0.47
Muslim	97(80.2)	90(74.4)	
Orthodox	16(13.2)	18(14.9)	
Protestant	8(6.6)	13(10.7)	
Maternal education			0.31
Illiterate	68(56.2)	59(48.8)	
Literate	53(43.8)	62(51.2)	
Maternal Occupation			0.01
Farmer	50(41.3)	38(31.4)	
Merchant	26(21.5)	14(11.6)	
House wife	45(37.2)	69(57.0)	
Father Occupation			0.73
Farmer	102(84.3)	99(81.8)	
Merchant	19(15.7)	22(18.2)	
Maternal age (in years)			0.63
20-24	6(5.0)	9(7.4)	
25-29	52(43.0)	43(35.5)	
30-34	31(25.6)	37(30.6)	
above 35	32(26.4)	32(26.4)	
Marital status			0.27
Married	110(90.9)	118(97.5)	
Divorced/Widowed	11(9.1)	3(2.5)	
Wealth index			0.02
1^st^ Quartile	35(28.9)	24(19.8)	
2^nd^ Quartile	36(29.8)	26(21.5)	
3^rd^ Quartile	29(24.0)	31(25.6)	
4^th^ Quartile	21(17.4)	40(33.1)	
Family size			0.02
2-4	24(19.8)	44(36.4)	
5-7	73(60.3)	60(49.6)	
8-10	24(19.8)	17(14)	
Children age (in months)			0.01
24-35	48(39.7)	30(24.8)	
36-47	48(39.7)	46(38.0)	
48-59	25(20.6)	45(37.2)	
Number of under 5 children			0.001
1	41(33.9)	66(54.5)	
2	60(49.6)	48(39.7)	
3	20(16.5)	7(5.8)	
Birth order			0.36
1-3	53(43.8)	61(50.4)	
Above 3	68(56.2)	60(49.6)	

Study participants (children) living in households with eight to ten [Adjusted Odds Ratio (AOR) = 4.44, 95% CI: 1.65, 11.95] and five to seven [AOR = 2.97, 95% CI: 1.41, 6.29] family members were more likely to be stunted than those living in households with two to four family members. Similarly, children living in households with three under-five children [AOR = 3.77, 95% CI: 1.33, 10.74] were more likely to develop stunting than those living in households with one under-five child. Children whose mothers worked as merchants [AOR = 4.03, 95% CI: 1.60, 10.17] and farmers [AOR = 3.92, 95% CI: 1.89, 8.16] were more likely to be stunted than children whose mothers worked as house wives. Children who breast fed for less than two years [AOR = 5.61, 95% CI: 1.49, 11.08] were more likely to be stunted than those who breast fed for two or more years. Children who were exclusively breast fed for less than six months [AOR = 3.27, 95% CI: 1.21, 8.82] and greater than six months [AOR = 7.62, 95% CI: 1.80, 12.23] were more likely to develop stunting than children who were exclusively breast fed for the first 6 months. Children who bottle fed [AOR =3.30, 95% CI: 1.33, 8.17)] and fed by hand [AOR = 3.04, 95% CI: 1.46, 6.32] were more likely to be stunted than children who fed their complementary food using spoon/cup (Table 2).

**Table 2:** **Factors independently associated with stunting among children of age 24 to 59 months in Meskan district, Gurage Zone, South Ethiopia, March 2013**

Variable	Case	Control	Crude OR	Adjusted OR
	Nº (%)	Nº (%)	(95% CI)	(95% CI)
Family size				
2-4 (ref.)	24(19.8)	44(36.4)	-	-
5-7	73(60.3)	60(49.6)	2.231(1.22, 4.08)**	2.97(1.41, 6.29)**
8-10	24(19.8)	17(14)	2.59(1.17, 5.74)*	4.44(1.65, 11.95)**
Number of under 5 children				
1 (ref.)	41(33.9)	66(54.5)	-	-
2	60(49.6)	48(39.7)	2.01(1.17, 3.47)*	1.88(0.97, 3.61)
3	20(16.5)	7(5.8)	4.60(1.79, 11.83)**	3.77(1.33, 10.74)*
Maternal Occupation				
Farmer	50(41.3)	38(31.4)	2.018(1.15, 3.55)**	3.92(1.89, 8.16)**
Merchant	26(21.5)	14(11.6)	2.85(1.35, 6.03)*	4.03(1.60, 10.17)**
House wife (ref.)	45(37.2)	69(57.0)	-	-
Duration of breast feeding				
< 24 months	16(13.2)	4(3.3)	4.46(1.44, 10.8)**	5.61(1.49, 11.08)*
≥ 24 months(ref.)	105(86.8)	117(96.7)	-	-
Duration of EBF				
For 6 months (ref.)	86(71.1)	105(86.8)	-	-
Below 6 months	21(17.4)	13(10.7)	1.972(0.93, 4.17)	3.27(1.21, 8.82)*
Above 6 months	14(11.6)	3(2.5)	5.69(1.58, 10.5)**	7.62(1.80, 12.23)**
Method of feeding				
Spoon & Cup (ref.)	50(41.3)	77(63.6)	-	-
Bottle	29(24.0)	14(11.6)	3.190(1.54, 6.62)**	3.30(1.33, 8.17)**
Hand	42(34.7)	30(24.8)	2.16(1.19, 3.88)**	3.04(1.46, 6.32)**

Variables included in the adjusted model are wealth index, child age, diarrhea, ARI, type of complementary food, number of food group, delivery attendance, mother hand washing practice, source of drinking water and maternal knowledge.

*p < 0.05, **p < 0.01, (ref.) - reference category.

## Discussion

This study intended to identify the factors associated to stunting among children age of 24 to 59 months using analytic study design. However, certain limitations may arise in the study such as recall bias and absence of data on: maternal nutrition, heights of the mothers, household food security and parasitic infections. There may be also misclassification of case and control, because of it is very difficult to get accurate age in country like Ethiopia. Efforts were made to get accurate age by asking for immunization card of the participants during the census. Lastly, since case-control study design was employed, it does not enable to establish temporality.

In this study participants living in households with high number of under-five children were about 4 times more likely to develop stunting than those living in households with least number of under-five children. This is consistent with community based studies conducted in southern Brazil and South Africa [12, 17]. Similarly children living in households with eight to ten family members were 4.44 and living in household with five to seven family members were 2.97 times more likely to develop stunting compared with those living in household with two to four family members. This might be due to resource depletion which exposed to poverty and decrement in food availability and also suggesting that there is more competition for available food when the household is large. This is in line with studies conducted in Northeastern Brazil, South Africa and Ethiopia [13, 17, 18]. But which is inconsistent with study conducted in Uganda [30].

Children whose mothers worked as merchant and farmer were more likely to develop stunting than children whose mothers worked as house wives. This might be due to decreased contact time to the child that brings short period of exclusive breast feeding, early cessation of breast feeding, increase exposure to bottle feeding and improper complementary food, which may have a large negative effect on the growth children. Our results are not consistent with studies that had shown a protective effect of maternal employment by increasing income and female autonomy. Employment may also positively influence food security, quality of diet and use of health services [21]. The difference is probably due to the fact that factors like child caring practice were overlooked in the previous study.

The likelihood of stunting was` higher among children who exclusively breast fed below or above the age of six months compared with who exclusively breast fed for six months. This finding was in line with study conducted different areas [17, 20, 23]. Inappropriate timing for introducing some kinds of complementary food to a child may affect his/her nutritional status because his/her digestive and immune systems are not yet mature. Introducing supplements before earlier, especially under unhygienic conditions, could be an important cause of malnutrition. On the other hand some studies demonstrated that association was not observed [22, 30]. The difference is probably due to difference in study design as well as geographical difference.

Our study showed that feeding complementary food using bottle and by hand increases the risk of stunting among children. This finding is consistent with other studies conducted in our country [23] in which feeding using bottle and hand were associated with increase the odds stunting.

## Conclusion

This study showed number of under-five children, family members, maternal occupation, duration of breast feeding, duration of exclusive breast feeding & method of feeding complementary food were independently associated with stunting. Thus, national public health intervention programmer and stakeholder working on improving child nutrition should focus on these determinants to reduce stunting. Health extension workers shall educate mothers/caretakers on the importance of exclusive breast feeding, methods of complementary feeding and adequate spacing between children. Program planner and policy makers should consider & strengthen collaboration and coordination of nutritional program that aimed to alleviate nutritional deficiencies and family health program.
